# Retraction: Proxalutamide Reduces the Rate of Hospitalization for COVID-19 Male Outpatients: A Randomized Double-Blinded Placebo-Controlled Trial

**DOI:** 10.3389/fmed.2022.964099

**Published:** 2022-06-10

**Authors:** Frontiers Editorial Office

The journal and Chief Editors have retracted the 19 July 2021 article cited above.

Shortly after publication, our office received letters of complaint questioning the integrity of the article, following which an Expression of Concern was published and a thorough investigation was conducted, in accordance with our policies and COPE guidelines.

The investigation found that the claims made in the conclusions were not adequately supported by the methodology of the study. In particular, as confirmed by an external expert, the process of allocation to treatment and control was not sufficiently random.

This retraction was approved by the Chief Editors of Frontiers in Medicine and the Chief Executive Editor of Frontiers. The authors disagree with this retraction.

